# Management of tubal ectopic pregnancy with methotrexate in the setting of symptomatic Coronavirus disease 2019 (COVID-19): A case report

**DOI:** 10.52054/FVVO.13.3.030

**Published:** 2021-09-24

**Authors:** NM Millan, J Morano, L Florez, J Carugno, CA Medina

**Affiliations:** Department of Obstetrics, Gynecology, and Reproductive Sciences, University of Miami, Health Systems, Jackson Memorial Hospital, Miami, Florida.

**Keywords:** Methotrexate, ectopic pregnancy, coronavirus

## Abstract

**Background:**

Coronavirus Disease 2019 (COVID-19) represents a complex therapeutic challenge. As the pandemic progresses, patients are presenting with ectopic pregnancies (EPs) and symptomatic COVID-19.

**Objective:**

We present the management of a patient with multiple medical comorbidities and tubal EP in the setting of severe symptomatic COVID-19 infection where all management options were precluded.

**Methods:**

Case report with literature review of management of tubal EP in the setting of severe symptomatic COVID-19 infection.

**Result:**

After careful consideration of options, the patient underwent successful medical management with methotrexate while receiving supportive care for COVID-19.

**Conclusions:**

Methotrexate proved to be the safest therapeutic option in this patient. Management of patients with severe COVID-19 and gynaecologic emergencies should be individualised and carefully reviewed with evolving knowledge of COVID-19.

## Introduction

The global pandemic of COVID-19 is caused by infection with enveloped positive-stranded RNA virus, severe acute respiratory syndrome coronavirus 2 (SARS-CoV-2) and may have multiple clinical presentations. Over 80% of patients infected with COVID-19 have mild disease, 14% severe illness, and 5% critical illness with an overall case-fatality rate of 2.3% among those with confirmed illness (Wu and McGoogan, 2020). Older age and medical comorbidities increase the risk for severe illness.

Ectopic pregnancy (EP) is an unfortunate condition occurring in 2% of pregnancies (ACOG, 2018). When untreated, rupture of the ectopic implantation site may occur, leading to haemodynamic instability and the need of emergengy surgical intervention. When identified early and if not ruptured, patients may be offered expectant, medical, or surgical intervention (American College of Obstetricians and Gynecologists’ (ACOG) Committee on Practice., 2018).

As the pandemic progresses, patients are presenting with EPs and symptomatic COVID-19 infection. Consideration of pulmonary comorbidity in the setting of active COVID-19 infection and development of pregnancy may preclude established medical regimens to treat an EP. We present the management of EP with methotrexate (MTX) in the setting of severe symptomatic COVID-19 infection where established treatment regimens were guarded.

## Case

A 36-year-old Gravida 6 Para 3 at 6 weeks 4 days of gestational age presented to the emergency room of our institution in July of 2020. The patient had history of asthma, chronic hypertension, and morbid obesity class III with a body mass index of 55 kg/m^2^. She presented with complaints of worsening dyspnea and chest pain and was found to have COVID-19. She was started on low-flow nasal cannula oxygen therapy to maintain her oxygen saturation above 92%. She also had severe orthopnoea (not tolerating -15° of inclination). Chest X-ray showed a constellation of findings concerning for atypical/viral pneumonia. She was admitted to the medicine service for supportive therapy including oxygen and dexamethasone, albuterol as needed, and clinical monitoring. Her Beta-hCG (B-hCG) serum level was 3,838 mIU/ mL and a transvaginal ultrasound demonstrated an endometrial thickness of 4 mm, with no evidence of an intrauterine pregnancy (IUP), and a right adnexal mass with peripheral vascularity, highly suggestive of a right tubal EP. There was no free fluid noted on the ultrasound. At this time, the patient was experiencing significant COVID- 19 symptoms, which were exacerbated by her underlying asthma and morbid obesity.

Of note, three days before presenting to our ED, she presented to a different hospital due to vaginal bleeding and abdominal pain. Her B-hCG serum level then was 2,359 mIU/mL and ultrasound failed to identify an IUP. She was discharged with precautions and follow-up for pregnancy of unknown location.

**Figure 1:**
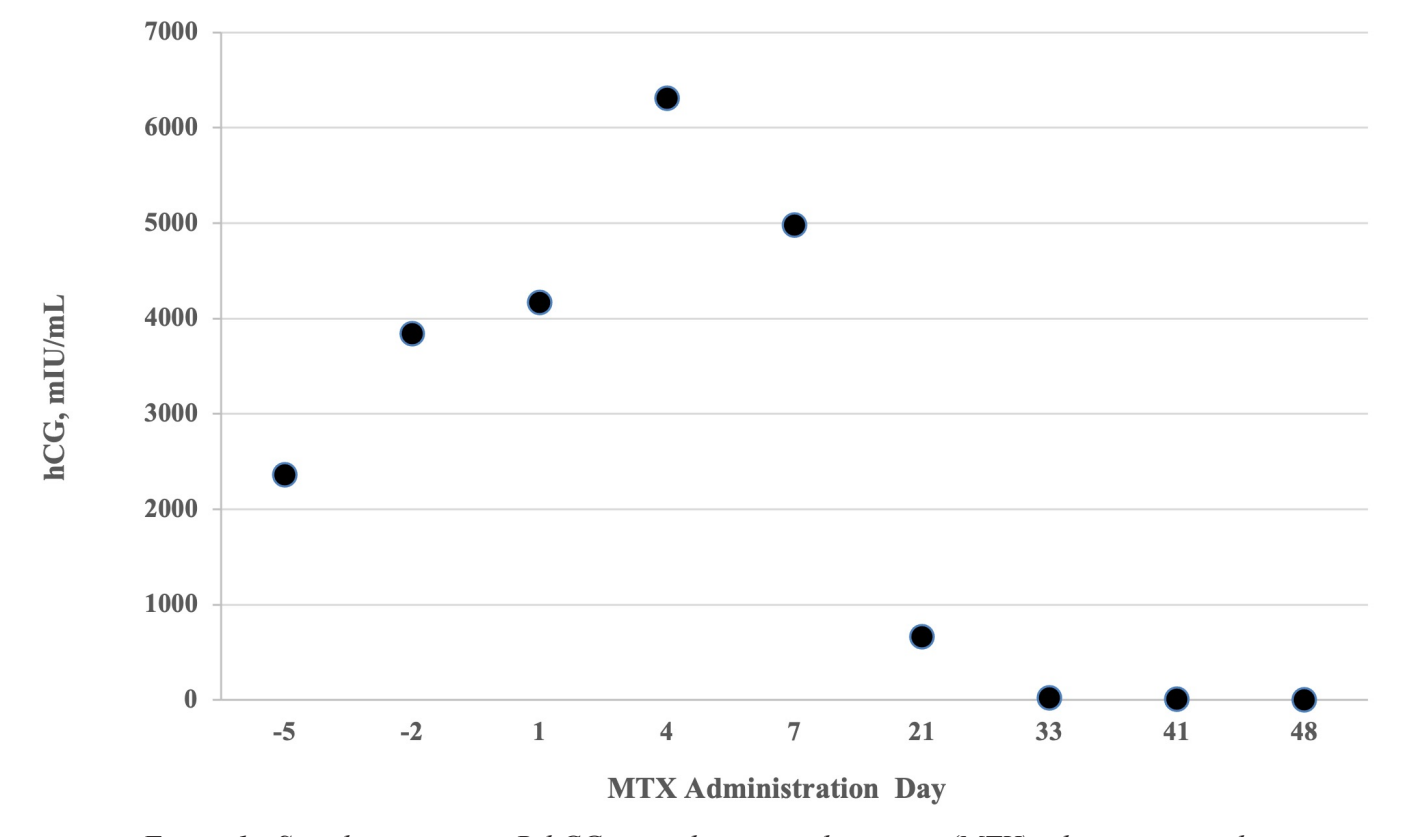
Serial quantitative B-hCG according to methotrexate (MTX) administration day. MTX administered days 1 and 4.

Considering the patient’s symptoms, active pulmonary disease, and comorbidities, a medical management of EP was initiated. A two-dose MTX protocol was carried out, with 50 mg/m 2 via intramuscular injection of MTX administered on hospital day (HD) 2 and 6. An appropriate drop in hCG >15% was noted (Figure 1). Mild transaminitis was observed following MTX therapy. The patient improved clinically and was stable for discharge by HD 10. She received outpatient follow-up with serial hCG measurements until non-pregnant levels were achieved. The patient provided written consent for publication of this case.

## Discussion

EP in the setting of symptomatic COVID-19 infection represents a challenge for the health care team. To the best of our knowledge, this is the first case report detailing EP management in a patient with severe COVID-19 respiratory symptoms. Hansel and Stovall recently published a case report of a patient with asymptomatic COVID-19 who was diagnosed with a non-ruptured EP (Hansen and Stovall, 2020). The authors emphasised evaluation of risks and benefits for each possible intervention and collaborative treatment decisions based on attainable limitations of disease progression and infectious spread. This underscored the focus of management of our patient’s EP.

In our case, extensive efforts between a multidisciplinary team were made to examine how her comorbidities would affect the management of the EP. Different therapeutic options were considered. Expectant management of an EP is an option for patients who meet specific criteria: 1) low and either decreasing or plateauing serum B-hCG levels, 2) asymptomatic, and 3) understand risk of potential need for surgical intervention if the EP ruptures (American College of Obstetricians and Gynecologists’ (ACOG) Committee on Practice., 2018). Success of expectant management has been reported in up to 88% of patients with B-hCG values less than 200 IU/L with decreasing success rates <25% for higher values of 2,000 IU/L (Korhonen et al., 1994). There are few small, randomised trials comparing efficacy of medical management with MTX compared to expectant management of EP for serum B-hCG levels under 2,000 IU showing no significant benefit of medical to expectant management (van Mello et al., 2013; Jurkovic et al., 2017).

Expectant management in this patient’s case presented was not possible given serially increasing B-hCG serum levels, symptoms, and unacceptable higher likelihood of failure.

The possibility of rupture resulting in hemodynamic instability requiring emergency surgery would be detrimental in this patient. She was deemed a high-risk surgical candidate due to her compromised respiratory status as a result of COVID-19 infection and underlying comorbidities of asthma and morbid obesity, which increased the chance of potential surgical/anaesthesia complication. She was strongly considered to be a difficult extubation possibly requiring long-term ventilator support postoperatively.

Additionally, increased morbidity and mortality are associated with performing surgery on patients with pulmonary compromise due to COVID-19. The COVIDSurg Collaborative demonstrated 51% of patients with COVID-19 who underwent a variety of surgical procedures experienced pulmonary complications with a 30-day mortality of 38% (COVIDSurg Collaborative, 2020). More concerning for our patient, the overall mortality reported was higher after emergency (26%) versus elective surgery (19%) (COVIDSurg Collaborative, 2020). Although not specified if symptomatic patients had worse surgical outcomes, it was strongly believed our patient’s respiratory symptoms would increase the morbidity and potential mortality associated with surgery.

Although uncertain with regards to transmission, consideration was also raised regarding exposure of healthcare personnel to viral particles in the operating theatre within escaping surgical smoke or during intubation or extubation (Saridogan and Grimbizis, 2020).

Lastly, medical management with chemotherapeutic agent MTX was considered. Others have previously suggested medical management as a theoretical option for COVID- 19 patients (Elito Júnior and Araujo Junior, 2020). Current management guidelines list active pulmonary disease and immunodeficiency as contraindications to the use of MTX (ACOG, 2018). However, this case warranted reconsideration of guidelines, focusing on patient-specific risks/ benefits of MTX therapy. Other concerns included selecting the appropriate MTX protocols and failure risk.

Most common side effects with MTX therapy are gastrointestinal, however, other rare side effects described are pulmonary toxicity, immunomodulation, and myelosuppression. Although not well understood, MTX-induced lung- injury has occurred via pulmonary hypersensitivity reaction (Cooper et al., 1986; Lynch and McCune, 1997), direct toxicity (Ohbayashi et al., 2010), activation of latent viral infection (Kim et al., 2009a, 2009b; St Clair et al., 1985;Golden et al., 1995), or idiosyncratic mechanism. These events may lead to pneumonitis, fibrosis, or pleuritis potentially worsening existing pulmonary disease as in our patient.

Patients with COVID-19 infection are noted to have leukocytosis and lymphopenia (Huang et al., 2020). Our patient initially had lymphopenia with an absolute lymphocyte count <1.0x103/mcL. There was concern MTX would worsen the immunosuppression and prolong or worsen the disease.

Adverse side effects are more likely with increased dose and duration of MTX. Theoretically, patients with severe symptomatic COVID-19 infection have a higher likelihood of developing side effects if renal function is impaired. Given the intermediate-dosage, limited exposure, and normal renal function in our patient, the risk of MTX toxicity and adverse effects was low.

Evidence shows comparable and not statistically different rates of resolution for EP between single- and multiple-fixed dose regimens (relative risk [RR], 1.07; 95% CI, 0.99–1.17) as well as single- versus two-dose therapeutic regimen (RR, 1.09; 95% CI 0.98–1.20) (Yang et al., 2017). The multiple fixed, versus the single-dose regimen would increase risk of the patient experiencing adverse effects by 64% (Yang et al., 2017). Meanwhile, the risk of developing side effects from two-dose regimen was 33% higher versus single-dose therapy (RR, 1.33; 95% CI, 0.92–1.94) (Yang et al., 2017).

Although, no cut-off value for serum B-hCG level has been determined to contraindicate medical management, increasing MTX therapy failure rates are reported with rising levels of pre- treatment serum B-hCG levels (American Society for Reproductive Medicine, 2013; Beguin et al., 2020). One study suggests the two- versus single- dose regimen is more favourable in patients with pre-treatment serum B-hCG levels between 3,600- 5,500 mIU/mL with successful resolution 88.9% versus 57.9% (P=0.03), respectively (odds ratio 5.80; 95% CI, 1.29–26.2) (Hamed et al., 2012).

Therefore, in our case, a two-dose approach was used to maximize the opportunity of treatment success as this patient’s pre-treatment serum B-hCG value was 4,166.7 mIU/mL. It was concluded despite her comorbidities; this was the safest approach in managing her non-ruptured EP compared with risks posed by surgical and expectant management.

This case addressed unanswered questions regarding suitable therapeutic approaches for patients with symptomatic COVID-19 and EP. Expectant management was precluded due to risk for rupture and need for emergent intervention. Surgical intervention would have increased morbidity and mortality. In this case, MTX for EP management was successfully administered.

Further investigation is needed on most appropriate means to address EP within the spectrum of COVID-19 presentation. Treating this patient led us to consider the following regarding management. Expectant management with close follow-up should only be considered if there exists evidence of serially decreasing B-hCG levels suggestive of a failing pregnancy. When contemplating medical management, patients should receive evaluation by a multidisciplinary team to identify risk factors for treatment failure and toxicity as well as contraindications to MTX therapy. When considering contraindications, active pulmonary disease should be considered within the entire spectrum of COVID- 19 presentation as asymptomatic patients may also present with underlying pathology and atypical imaging (Hu et al., 2020). The COVID-19 disease process may theoretically be worsened by MTX administration. Based on our limited findings, however, it may be considered in patients without other alternatives. A plan should be devised prior to intervention to respond to change of patient status as needed.

If surgical intervention is needed, the patients should be evaluated by all relevant care teams and medically optimised for the procedure. If general anaesthesia is contraindicated, alternative methods of sedation and analgesia may be considered. Efforts to limit exposure of healthcare personnel while effectively managing EP should be made, such as using minimally invasive technique with only necessary staff in the operating theatre. Personnel should wear personal protective equipment during any intervention.

Any patient with high suspicion for EP rupture, should have rapid COVID-19 testing and be taken to the operating theatre for emergency surgery. Delaying surgical intervention, when deemed appropriate is strongly discouraged, since the benefits outweigh the risk of increased morbidity/mortality.

Overall, patients presenting with EP and symptomatic COVID-19 need individualised and collaborative treatment planning. Patients should be extensively counselled regarding the risks of each management option. When available, multidisciplinary treatment planning should occur to reduce risk of morbidity/ mortality.
